# *Helicobacter pylori* induces a novel form of innate immune memory via accumulation of NF-кB proteins

**DOI:** 10.3389/fimmu.2023.1290833

**Published:** 2023-11-20

**Authors:** Tobias Frauenlob, Theresa Neuper, Christof Regl, Veronika Schaepertoens, Michael S. Unger, Anna-Lena Oswald, Hieu-Hoa Dang, Christian G. Huber, Fritz Aberger, Silja Wessler, Jutta Horejs-Hoeck

**Affiliations:** ^1^ Department of Biosciences and Medical Biology, University of Salzburg, Salzburg, Austria; ^2^ Cancer Cluster Salzburg (CCS), Salzburg, Austria; ^3^ Center for Tumorbiology and Immunology (CTBI), University of Salzburg, Salzburg, Austria

**Keywords:** *H. pylori*, trained immunity, innate immune memory, NF-кB, innate immunity, tolerance, monocytes, inflammation

## Abstract

*Helicobacter pylori* is a widespread Gram-negative pathogen involved in a variety of gastrointestinal diseases, including gastritis, ulceration, mucosa-associated lymphoid tissue (MALT) lymphoma and gastric cancer. Immune responses aimed at eradication of *H. pylori* often prove futile, and paradoxically play a crucial role in the degeneration of epithelial integrity and disease progression. We have previously shown that *H. pylori* infection of primary human monocytes increases their potential to respond to subsequent bacterial stimuli – a process that may be involved in the generation of exaggerated, yet ineffective immune responses directed against the pathogen. In this study, we show that *H. pylori*-induced monocyte priming is not a common feature of Gram-negative bacteria, as *Acinetobacter lwoffii* induces tolerance to subsequent *Escherichia coli* lipopolysaccharide (LPS) challenge. Although the increased reactivity of *H. pylori-*infected monocytes seems to be specific to *H. pylori*, it appears to be independent of its virulence factors Cag pathogenicity island (CagPAI), cytotoxin associated gene A (CagA), vacuolating toxin A (VacA) and γ-glutamyl transferase (γ-GT). Utilizing whole-cell proteomics complemented with biochemical signaling studies, we show that *H. pylori* infection of monocytes induces a unique proteomic signature compared to other pro-inflammatory priming stimuli, namely LPS and the pathobiont *A. lwoffii*. Contrary to these tolerance-inducing stimuli, *H. pylori* priming leads to accumulation of NF-кB proteins, including p65/RelA, and thus to the acquisition of a monocyte phenotype more responsive to subsequent LPS challenge. The plasticity of pro-inflammatory responses based on abundance and availability of intracellular signaling molecules may be a heretofore underappreciated form of regulating innate immune memory as well as a novel facet of the pathobiology induced by *H. pylori*.

## Introduction

*Helicobacter pylori* is a Gram-negative pathogen infecting approximately 50% of the World’s population (1). Due to its malignancy-promoting properties, this prokaryote has been designated by the International Agency for Research on Cancer as a class I carcinogen (2). Extensive inflammation of the gastric epithelium is a hallmark of *H. pylori* infection and is assumed to be one of the main drivers of gastric carcinogenesis. Immune cells migrating into the gastric epithelium, recruited from peripheral blood or adjacent lymphoid structures (such as mucosa-associated lymphoid tissue), produce a variety of pro-inflammatory cytokines and chemoattractant molecules, which increase the influx of additional immune cells (3, 4). This vicious cycle may lead to severe consequences, including atrophy of the gastric epithelium and manifestation of ulcers and neoplastic lesions (5), which are a result of overt immune activation, among other factors. *H. pylori*, however, has proven resourceful in subjugating immune reactions and employing numerous virulence factors to evade host immunity and chronically colonize the gastric epithelium (6–9). These virulence factors interfere with vital processes involved in pattern recognition receptor signaling and downstream signal transduction. The most well-known is cytotoxin-associated gene A (CagA), an effector molecule injected into eukaryotic host cells by the bacterial type IV secretion system (T4SS) (10, 11). Both T4SS and CagA are encoded by the genetic element termed the Cag pathogenicity island (CagPAI). Other virulence factors, including vacuolating toxin A (VacA) and γ-glutamyl-transferase (γ-GT), have previously been implicated in the subversion of immunity (12, 13).

A lesser understood, albeit highly intriguing facet of immunobiology is the gastric pathogen’s effect on innate immune memory. A variety of pathogen-associated molecular patterns and bacterial pathogens, but also host-derived molecules, have already been reported to influence the innate immune system’s capacity to “remember” past encounters and adapt subsequent responses accordingly, leading to either an increase (i.e., training) or a decline (tolerance) in reactivity to later challenge (14–17). These adaptations may be beneficial or detrimental to the host, as maladaptive, unchecked inflammatory activity can have severe consequences, including tissue degeneration and loss of function (18, 19). *H. pylori* infection severely disrupts homeostatic processes, and its pathology is thought to be largely due to the ensuing inflammatory response, the etiology of which is not entirely clear. We previously tested the hypothesis that human primary monocytes undergo specific phenotypic alterations after priming with *H. pylori* (20), leading to modulation of their activity. Our preliminary analysis indeed revealed an *H. pylori*-mediated priming effect on monocytes, which, contrary to other trained immunity effects, leads to an acute surge of reactivity to a subsequent *Escherichia coli* lipopolysaccharide (LPS)-challenge, rather than requiring a typical adaptation period of several days. This temporal aspect is particularly intriguing, as it is widely known that pro-inflammatory stimuli, such as *E. coli* LPS, Pam3CSK4 or flagellin (FLA) (21), primarily elicit immediate tolerance (i.e., “endotoxin tolerance”) to subsequent challenge.

Here, we corroborate and extend our previous findings by highlighting the uniqueness of *H. pylori* in its ability to induce a hyperreactive phenotype in human primary monocytes in comparison to other pro-inflammatory molecules and bacterial stimuli. Combining proteomics with conventional molecular biology techniques, we find that *H. pylori* priming strongly induces the accumulation of NF-кB subunits in human primary monocytes. Moreover, we establish that the increased availability of NF-кB proteins correlates with the reactivity of cells to subsequent LPS challenge. In contrast, monocytes primed with other pro-inflammatory stimuli (LPS and the bacterial pathobiont *Acinetobacter lwoffii*) were refractory to challenge both in their capacity to induce active signaling downstream of the LPS receptor Toll-like receptor 4 (TLR4) as well as in their ability to produce tumor necrosis factor α (TNF-α). Taken together, we describe a previously unknown mechanism via which *H. pylori* increases abundance of intracellular signaling molecules in innate immune cells, leading to hyperresponsiveness to subsequent challenge.

## Methods

This study was conducted in accordance with established guidelines of the World Medical Association’s Declaration of Helsinki. As Austrian national regulations do not require additional consent from anonymous blood donors for scientific use of blood cells discarded after leukapheresis, no further approval of the study by the local ethics committee was required.

### Bacterial culture

*Helicobacter pylori* P12 and respective genetic mutants (ΔCagA, ΔCagPAI, ΔVacA, Δγ-GT) were cultured under microaerophilic conditions using Oxoid 3.5 L buckets (Thermo Fisher Scientific, Vienna, Austria, no longer available, replaced by AnaeroBox 3.5 L, #AB0035L) and Oxoid CampyGen 3.5 L sachets (Thermo Fisher Scientific, #CN0035A) on GC agar plates containing 10% horse serum (Biowest, Nuaillé, France, #S0910) and selective antibiotics, if appropriate. Bacteria were allowed to grow over the course of 72 h and then re-plated and cultured overnight prior to infection of eukaryotic cells. *Acinetobacter lwoffii* (DSM 2403) was plated the day before infection on nutrient broth plates and allowed to grow overnight. In advance of infection, bacteria were harvested from plates with cotton buds (Paul Boettger, Bodenmais, Germany, #09-129-0100) and solubilized in 1 mL phosphate-buffered saline (PBS) solution (Sigma-Aldrich, Vienna, Austria, #P2272). Bacterial density was then determined by spectrophotometric measurement at OD600 on a BioPhotometer Plus device (Eppendorf, Vienna, Austria, #6132) and the bacterial cell count was determined via an in-house calibration curve. Multiplicity of infection (MOI) of 5 was used throughout all experiments.

### Cell isolation, culture, stimulation and lysis

Human primary monocytes were isolated from fresh buffy coats of blood donations provided by healthy, anonymized volunteers obtained from the Blood Bank Salzburg. Peripheral blood mononuclear cells were isolated via gradient density centrifugation using HistoPaque-1077 (Sigma-Aldrich, #10771). Monocytes were then magnetically labeled using CD14 MicroBeads (Miltenyi Biotec, Bergisch-Gladbach, Germany, #130-050-201) and purified according to the manufacturer’s instructions. Monocytes were then cultured in RPMI-1640 medium (Sigma-Aldrich, #R0883) supplemented with 10% heat-inactivated fetal calf serum (Biowest, #S1400) and 1% L-glutamine (Fisher Scientific, Schwerten, Germany, #11539876). After isolation, monocytes were incubated between 1 and 4 h at 37°C or at 4°C overnight until further use. For mRNA and protein expression studies, monocytes were seeded at a density of 2×10^5^ to 1×10^6^ cells/mL and stimulated with *E. coli* LPS (O55:B5, Sigma-Aldrich, #L2880) at a final concentration of 5 ng/mL as well as *H. pylori* and *A. lwoffii* at MOI 5 for the indicated period. For mRNA expression analysis, cells were harvested in TRI reagent (Sigma-Aldrich, #93289), whereas for protein expression studies, cells were lysed in cell lysis buffer II (Fisher Scientific, #FNN0021) supplemented with 0.1 mM PMSF (Sigma-Aldrich, #93482) and cOmplete Mini EDTA-free protease inhibitor cocktail (Roche, Vienna, Austria, #04693159001), according to the manufacturer’s instructions. Cell lysates for Western blot analysis were centrifuged for 15 min at 15000×*g* at 4°C and then frozen at -20°C. Protein concentration of lysates was determined by Pierce BCA assay (Thermo Fisher Scientific, #23225), followed by dilution of lysates with 6× reducing Laemmli sample buffer (Thermo Fisher Scientific, #J61337.AC) and heating of samples at 95°C for 5 min.

### Monocyte innate immune memory model

For innate immune memory experiments, monocytes were stimulated as previously described (20). Briefly, monocytes were seeded at a concentration of 1×10^6^ cells/mL in 100 µL medium in 96-well flat bottom cell culture plates (Corning, Kaiserslautern, Germany, #3585) and rested for 1 h. Then, in nuclear factor erythroid 2-related factor 2 (NRF2) inhibition experiments, NRF2 inhibitor ML-385 (Sigma-Aldrich) was added in 50 µL to achieve a final concentration of 10 µM and cells were rested for an additional 30 min. Subsequently, 50 µL of control medium, medium containing *E. coli* LPS (O55:B5, Sigma-Aldrich, final concentration of 5 ng/mL), *H. pylori* or genetic mutants or *A. lwoffii* was added to the respective wells to reach a final volume of 200 µL. In experiments where no inhibitor was used, the stimulation agents were added in 100-µL volumes instead of 50 µL. Initial stimulation (priming) occurred for 24 h, after which the supernatants were carefully removed from each well, leaving the adherent monocytes unperturbed at the bottom of the well. Immediately after, 200 µL of fresh medium containing 1% Pen/Strep ± 10 ng/mL LPS (challenge or control) or 10 ng/mL LPS and NF-кB inhibitor BAY 11-7082 (Sigma-Aldrich) at a final concentration of 10 µM were added. Monocytes were then cultured for a further 24 h and supernatants collected for analysis.

For testing responsiveness of primed monocytes to challenge and subcellular fractionation, 1.5×10^6^ monocytes were seeded in a 6-well plate (Corning, #3506) and primed as described above for 24 h. Thereafter, cells were transferred into 1,5 mL tubes (Eppendorf, #0030125150), centrifuged at 300×*g*. The supernatant was subsequently discarded, and the cells were resuspended in challenge medium containing 1% Pen/Strep (Sigma-Aldrich, #P4333) ± 10 ng/mL LPS and incubated at 37°C, 5% CO_2_ for the indicated time frame (0 min, 15 min, 30 min, 60 min; 30 min for subcellular fractionation). Then, cells were pelleted again and lysed as described above for analysis of intracellular signaling molecule phosphorylation status via Western blot. For subcellular fractionation and analysis of nuclear and cytoplasmic protein contents, Cell Fractionation Kit (Cell Signaling Technology, Frankfurt, Germany, #9083) was used according to the manufacturer’s instructions.

For testing of innate immune memory induction to other bacterial and pro-inflammatory mediators, the following molecules and concentrations were used: flagellin (FLA) from *Pseudomonas aeruginosa* (Invivogen, Toulouse, France, #tlrl-pafla), 50 ng/mL during priming, 100 ng/mL during challenge; human recombinant TNF-α (ImmunoTools, Friesoythe, Germany, #11343013), 5 ng/mL during priming.

### SDS-PAGE and Western blot

Purified and denatured cell extracts were separated by SDS-PAGE on a 4-12% Bis-Tris gel (Thermo Fisher Scientific, Vienna, Austria, #NP0321BOX, #NP0322BOX) and transferred onto nitrocellulose membranes (0.45 µm pore size, Bio-Rad, Vienna, Austria, #1620115) using a Trans-Blot transfer cell (Bio-Rad). Thereafter, nonspecific binding sites were blocked by incubation with 5% (w/v) non-fat powdered milk (Roth, Karlsruhe, Germany, #T145.1) in 50 mM Tris-HCl pH 7.6, 150 mM NaCl and 0.1% (v/v) Tween-20 (Sigma-Aldrich, #P1379) (TBS-T) for 1 h at room temperature (RT). The membrane was then incubated with the primary antibody diluted in 5% bovine serum albumin (Roth, #1003336101) (w/v) in TBS-T overnight on a shaker at 4°C. The blots were then washed 3 times for 5 min with TBS-T before being incubated with horseradish-peroxidase-labeled secondary anti-rabbit IgG for 1 h at RT. Membranes were then washed a further 3 times for 5 min and incubated in West Pico PLUS chemiluminescent substrate (Thermo Fisher Scientific, #34579) prior to detection by a ChemiDoc imaging device (Bio-Rad). The following primary (all rabbit) and secondary antibodies (goat) were used according to the manufacturer’s instructions (all from Cell Signaling Technology): β-Actin (13E5) #4970, Sequestosome-1 (SQSTM1/p62) (D1Q5S) #39749, heme oxygenase-1 (HO-1) (E3F4S) #43966, NADPH quinone oxidoreductase 1 (NQO1) (D6H3A) #62262, c-Rel (D4Y6M) #12707, p65 (RelA) (D14E12) #8242, NF-кB1 p105/p50 (D4P4D) #13586, NF-кB2 p100/p52 (D7A9K) #37359, phospho-specific IKKαβ (Ser176/180) (16A6) #2697, phospho-specific p65 (Ser536) (93H1) #3033, p44/42 MAPK (Erk1/2, used as housekeeping protein for cytoplasmic fraction of subcellular fractionation Western blots) (137F5) #4695, histone H3 (used as housekeeping protein for nuclear fraction of subcellular fractionation Western blots) (D1H2) #4499, anti-rabbit IgG, HRP-linked secondary antibody #7074. Western blots were quantified using Fiji (ImageJ1.54f).

### Whole-cell proteomic analysis and bioinformatics

Pelleted, primed monocytes (1.5×10^6^) were resolved in 60 µL of 50 mM triethylammonium bicarbonate buffer (TEAB, pH 8.50, Sigma-Aldrich, #T7408) containing 5% (w/w) SDS (Sigma-Aldrich, L3771) and 1× cOmplete Mini EDTA-free protease inhibitor cocktail (Roche, #04693159001). Subsequently the cells were lysed by heating for 5 min at 95°C, followed by sonication in a Bioruptor device (Diagenode, Liège, Belgium) for 5 min. After a 1-min centrifugation step at 14,000 g, the protein content in the supernatant was analyzed by a Pierce BCA Protein assay kit (Thermo Fisher Scientific, #23255) according to the manufacturer’s instructions. Next the lysates were treated with 5 mM tris-(2-carboxyethyl)-phosphine-hydrochloride (TCEP, Sigma-Aldrich, #75259) at 55°C for 15 min to reduce disulfides, followed by alkylation of the cysteine residues by addition of iodoacetamide (Sigma-Aldrich, #I6125) to a concentration 40 mM and incubation at 22°C in the dark for 10 min. Subsequently the samples were acidified to pH ≤1 with 12% (v/v) ortho-phosphoric acid (Merck, Darmstadt, Germany, #100573) followed by protein precipitation by adding 7:1 (v/v) of 100 mM TEAB (pH 7.55) in 90% methanol (v/v; Merck, #106009). Next the proteins were purified by suspension trapping employing S-Trap mini columns (Protifi, Huntington, NY, USA) according to the manufacturer’s instructions, and digested to peptides with trypsin (sequencing grade modified, porcine, Promega, Madison, WI, USA, #V5111) at a protease/protein ratio of 1:10 (w/w) at 37°C for 16 h. The obtained peptides were dried at 50°C in a vacuum centrifuge and resuspended in 100 mM TEAB (pH 8.5) to a concentration of 0.50 µg/µL. 10 µg of each sample were labeled employing the TMTpro™ 16plex kit (Thermo Fisher Scientific, #A44521, WF324547) according to the manufacturer’s protocol. A pool sample containing 0.83 µg of each sample was labeled with the 126C channel to serve as the reference channel. After quenching of the labeling reaction by addition of hydroxylamine to a concentration of 0.2% (v/v), all samples were pooled and dried at 60°C in a vacuum centrifuge. This was followed by resuspension in 100 µL 0.10% (v/v) aqueous trifluoracetic acid (TFA, Sigma-Aldrich, #80457) and purification of the peptides applying C18 zip-tips (Thermo Fisher Scientific, #87784) according to the manufacturer’s protocol. The purified peptides were dried once more at 50°C in a vacuum centrifuge before resuspension in an aqueous solution of 1% acetonitrile (ACN; VWR International, Vienna, Austria, #20060.320) and 0.1% formic acid (FA, Sigma-Aldrich, #1002631000) (v/v) to a final concentration of 3.3 µg/µL.

Chromatographic separation of 3.3 µg of peptides was carried out by employing reversed phase HPLC on an UltiMate™ 3000 RSLCnano System (Thermo Fisher Scientific, Germering, Germany), on a DNV PepMap™ Neo column (750 x 0.075 mm i.d.) (Thermo Fisher Scientific, #DNV75750PN). For the separation, 0.10% aqueous FA (solvent A) and 0.10% FA in ACN (solvent B) were pumped at a flow rate of 200 nL/min in the following order: 1.0% B for 5.0 min, a linear gradient from 1.0-10.0% B in 25 min, a second linear gradient from 10.0-35.0% B in 470.0 min, and a third linear gradient from 35.0-45.0% B in 95.0 min. This was followed by flushing with 99.0% B for 5 min and column re-equilibration with 1.0% B for 35 min. The column temperature was kept constant at 50°C, the autosampler was kept at 4°C.

The nanoHPLC system was hyphenated to a Q Exactive™ Hybrid Quadrupole-Orbitrap™ mass spectrometer via a Nanospray Flex™ ion source (both from Thermo Fisher Scientific, Bremen, Germany). The source was equipped with a SilicaTip emitter with 360 µm o.d., 20 µm i.d. and a tip i.d. of 10 µm purchased from CoAnn Technologies Inc. (Richland, WA, USA, #TIP36002010-12). The spray voltage was set to 1.5 kV, S-lens RF level to 60.0 and capillary temperature to 250°C. Each scan cycle consisted of a full scan at a scan range of *m/z* 350–2,000 and a resolution setting of 70,000 at *m/z* 200, followed by 15 data-dependent higher-energy collisional dissociation (HCD) scans in a 1.2 *m/z* isolation window at 30% normalized collision energy at a resolution setting of 35,000 at *m/z* 200. For the full scan the automatic gain control (AGC) target was set to 3e6 charges with a maximum injection time of 120 ms, for the HCD scans the AGC target was 2e5 charges with a maximum injection time of 250 ms. Already fragmented precursor ions were excluded for 30 seconds. Data acquisition was conducted using Thermo Scientific™ Chromeleon™ 7.2 CDS (Thermo Fisher Scientific).

For data evaluation, MaxQuant 2.0.1.0 (22) was used with the setting Reporter ion MS2, using weighted ratio to reference channel for normalization and correcting for isotope impurities in the TMTpro reagents. For protein identification, a database from the UniProt consortium (23) including only reviewed Swiss-Prot entries for *Homo sapiens* (Human) from 10.03.2023 was used applying a 1% false discovery rate. The obtained protein groups (MaxQuant output file “proteinGroups.txt”) were further processed using the Perseus 1.6.14.0 software platform (24): First the reporter intensities were log2 transformed, followed by the removal of proteins that were not detected in all samples, as well as the removal of proteins that were only identified by site, reverse sequence matches and potential contaminants like various isoforms of keratin. Next the reporter ion intensities were normalized by subtraction of the median intensity in each sample followed by a principal component analysis (PCA). As the PCA indicated a clustering not only dependent on the treatment, but also on the donor of the monocytes, the intensities were batch corrected employing the Remove batch effect function with the method *Limma* (25) embedded in the PerseusR plugin (26). Differential expression analysis was likewise performed with *Limma* employing R (version 4.2.2) in RStudio (version 2023.03.1 + 446), and p-value adjustment was conducted with the Benjamini-Hochberg procedure. Fast gene set enrichment analysis was performed using fgsea (27) package in R (version 4.3.0). The following databases were downloaded for the fgsea analysis: WikiPathways 2019 Human, NCI-Nature 2016, TRRUST Transcription Factors 2019, MSigDB Hallmark 2020, GO Cellular Component 2018, CORUM, KEGG 2019 Human, TRANSFAC and JASPAR PWMs, ENCODE and ChEA Consensus TFs from ChIP-X, GO Biological Process 2018, GO Molecular Function 2018, Human Gene Atlas. The mass spectrometry proteomics data have been deposited in the ProteomeXchange Consortium (http://proteomecentral.proteomexchange.org) via the PRIDE partner repository (28) with the dataset identifier PXD045072. All data analysis is freely accessible and can be found in the following GitHub repository: https://github.com/VSchaepertoens/monocytes_fgsea.

### Luminex assay

Cytokine and chemokine secretion was studied by bead-based multiplex assay using the Inflammation 20-Plex Human ProcartaPlex Panel (Thermo Fisher Scientific, #EPX200-12185-901). Beads were washed in PBS, 0.05% Tween-20 before being resuspended in Assay Buffer and distributed in 96-well V-bottom plates (Greiner BioOne, Kremsmünster, Austria, #651101). Standards or samples were incubated with beads overnight on an orbital shaker at 4°C. The plate was then washed 3 times with 150 µL wash buffer per well and incubated with Detection Antibody solution for 30 min at RT on an orbital shaker. Thereafter, the plate was washed 3 times with 150 µL wash buffer per well and incubated with Streptavidin-PE solution for 30 min at RT on an orbital shaker. The plate was subsequently washed 3 times with 150 µL wash buffer and the well contents were resuspended in Reading Buffer. Quantification was performed on a Luminex MagPix device (Luminex, Austin, TX, USA) and data were analyzed via browser-based ProcartaPlex Analyst software.

### Immunofluorescence and confocal *microscopy*

For analysis of p65/RelA expression in primed human monocytes, 5×10^5^ cells were seeded and primed for 24 h as described above in 24-well plates. Carefully, the upper half of the supernatants were taken off and cells were infused and fixed with paraformaldehyde for 15 minutes at RT, using a final concentration of 3%. Then, cells were harvested from the wells and centrifuged for 5 minutes at 2000 rpm. Supernatants were discarded and cell pellets were resuspended in PBS. Cells were spun onto object slides via a Cytospin 4 centrifuge (Epredia, Dreieich, Germany) at 600 rpm for 5 minutes. Sample regions were marked with Hydrophobic Barrier Pap Pen (Thermo Fisher Scientific, #R3777) and then transferred to a wet staining chamber and washed two times with PBS. Cells were then permeabilized with 0.1% TritonX100 (Sigma Aldrich, #93443) for 10 minutes, followed by two washes with PBS. Unspecific binding sites were blocked with 2% BSA and 5% donkey serum (Sigma Aldrich, #D9663) for 1 h at RT. Samples were then incubated with primary rabbit anti-human p65/RelA antibody (Cell Signaling Technology, (D14E12) #8242, 1:400 dilution) overnight at 4°C. Thereafter, samples were washed extensively in PBS and incubated for 2 h at RT in the dark with secondary donkey anti-rabbit AF568 antibody (Invitrogen, #A10042, 1:1000 dilution) and nucleus counterstain 4’,6-diamidino-2-phenyl-indol-dihydrochloride (DAPI, Sigma Aldrich, #MBD0015, 1:2000 dilution). Cells were subsequently washed 3 times with PBS and semi-dry mounted with glass cover slips in Pro-Long Gold Antifade Mountant (Invitrogen, #P36934). For total p65/RelA protein expression analysis, monocytes were imaged with a 100x oil objective and the Olympus IX83 widefield fluorescence microscope. All images were taken with the same microscope settings. In total, three different images per experimental condition from four individual donors were analyzed (n=4/group). From each image, the number of cells was manually determined and the mean gray values = mean intensity (AU) for p65/RelA signals were analyzed using Fiji (ImageJ1.54f). For every image the mean intensity was divided by the number of cells and the average of three images was calculated. Data are shown as average p65/RelA intensities (AU) per cell.

Nuclear translocation of p65/RelA was analyzed by using a Zeiss Observer Z1 fluorescence microscope equipped with an Abberior Instruments STEDYCON unit for confocal and super-resolution STED microscopy. Confocal images were taken with a 100x oil objective (ROI: 60x60 µm) from single focal z-planes. Co-localization analysis of p65/RelA with the DAPI cell nucleus signal was performed using the JACoP plugin for Fiji (29). The thresholds for both signals were manually set for each image and the M1 (Manders’ coefficient, i.e., the fraction of p65/RelA overlapping with DAPI, range: 0-1) was calculated. In total, three different images per experimental condition from four individual donors were analyzed (n=4/group). The mean M1 values from three images and further the percentage values were calculated and are shown as % co-localization of p65/RelA with DAPI. All representative images were post-processed with Fiji (ImageJ1.54f) and Microsoft PowerPoint.

### ELISA

TNF-α (#900-T25) and interleukin-6 (IL-6, #900-T16) ELISAs (Peprotech, London, United Kingdom) were performed on supernatants according to the manufacturer’s instructions.

### Statistical analyses

Data are presented as symbols representing individual donors with bars indicating the mean. Statistical analyses were performed via GraphPad Prism 10 Software (GraphPad Software, San Diego, CA, USA). Differences between multiple stimulation groups were analyzed via one-way ANOVA including appropriate *post-hoc* tests. Sample size is indicated in the figure legends. *p* values < 0.05 were deemed significant (*p ≤ 0.05, **p ≤ 0.01, ***p ≤ 0.001, ****p ≤ 0.001).

## Results

### Monocyte hypersensitivity to *E. coli* LPS is explicitly induced by *H. pylori* but does not require its characteristic virulence factors CagPAI, CagA, VacA and γ-GT

**Figure 1:**
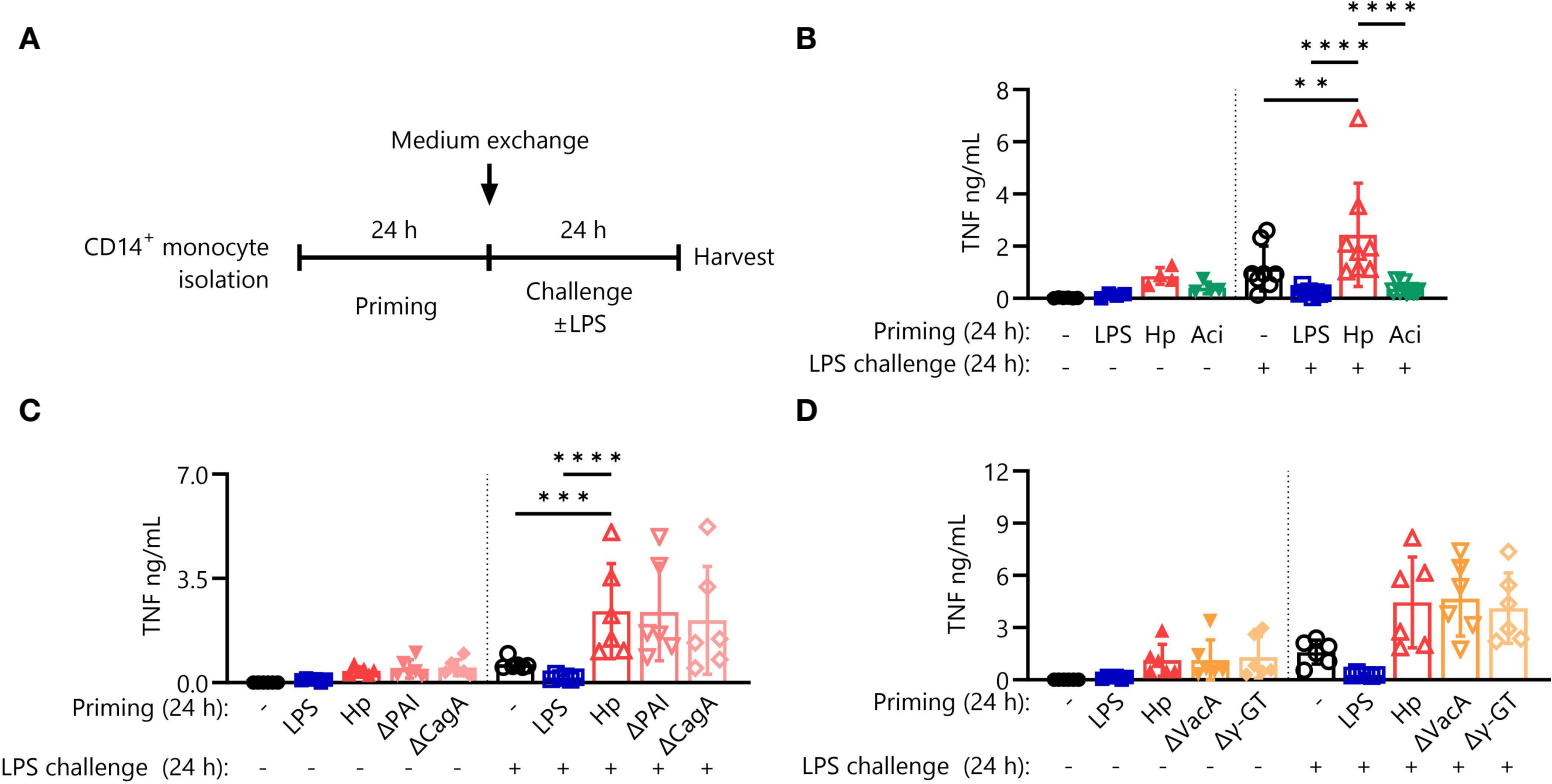
Hypersensitivity to LPS after priming is specific for *H. pylori* but not incited by prototypical virulence factors. **(A)** Experimental setting of monocyte short-term innate immune memory model. Monocytes were primed with *E*. *coli* LPS (5 ng/mL), *H*. *pylori* (Hp) or respective mutants, *A*. *lwoffii* (Aci) (all MOI 5) or remained unstimulated for 24 (h) Then the medium was exchanged to challenge medium, either containing LPS (10 ng/mL) or not. **(B)** TNF-α secretion by monocytes primed with LPS (5 ng/mL), *H*. *pylori*, *A*. *lwoffii* (both MOI 5) or unprimed cells challenged, or not, with LPS (10 ng/mL) was measured via ELISA. **(C, D)** TNF-α secretion of monocytes primed with LPS, Hp WT or respective mutants or unprimed cells challenged, or not, with LPS was measured via ELISA. Bars represent mean ± SD of six to eight individual donors and at least two independent experiments. For statistical analysis, RM-ANOVA with Šidak’s *post-hoc* test was performed (**p ≤ 0.01, ***p ≤ 0.001, ****p ≤ 0.0001).

As described previously, priming of primary human monocytes with *H. pylori* induces hyperresponsiveness to subsequent challenge with *E. coli* LPS (hereafter referred to as LPS) (20). LPS priming, on the other hand, effectively shuts down production of the pro-inflammatory cytokine TNF-α in response to LPS challenge. To understand if monocyte hyperresponsiveness to challenge is a common feature of live bacterial priming stimuli, we extended our experiments to include the opportunistic Gram-negative pathobiont *A. lwoffii*, according to the experimental scheme depicted in **Figure 1A**. In contrast to the priming effect after *H. pylori* stimulation, *A. lwoffii*-primed monocytes developed strong tolerance (**Figure 1B**), akin to the effects of other pro-inflammatory molecules when used as priming agents, such as flagellin and TNF-α (**Supplementary Figure S1**). Thus, having determined that immediate hyperresponsiveness to LPS challenge is not elicited by priming with another bacterial species, we next asked if *H. pylori*-intrinsic factors are responsible for the priming effect. To investigate the potential contribution of well-defined *H. pylori* virulence factors to the above-described hyperresponsiveness to LPS challenge, we primed monocytes with *H. pylori* mutant strains devoid of the virulence factors CagPAI and CagA (**Figure 1C**), as well as VacA and γ-GT (**Figure 1D**). These mutant strains elicited essentially equal levels of TNF-α production in response to LPS challenge compared to priming with *H. pylori* WT (**Figures 1C, D**), indicating no significant contribution of the examined virulence factors to *H. pylori*-induced monocyte priming.

### Different priming stimuli induce distinct proteomic signatures

**Figure 2:**
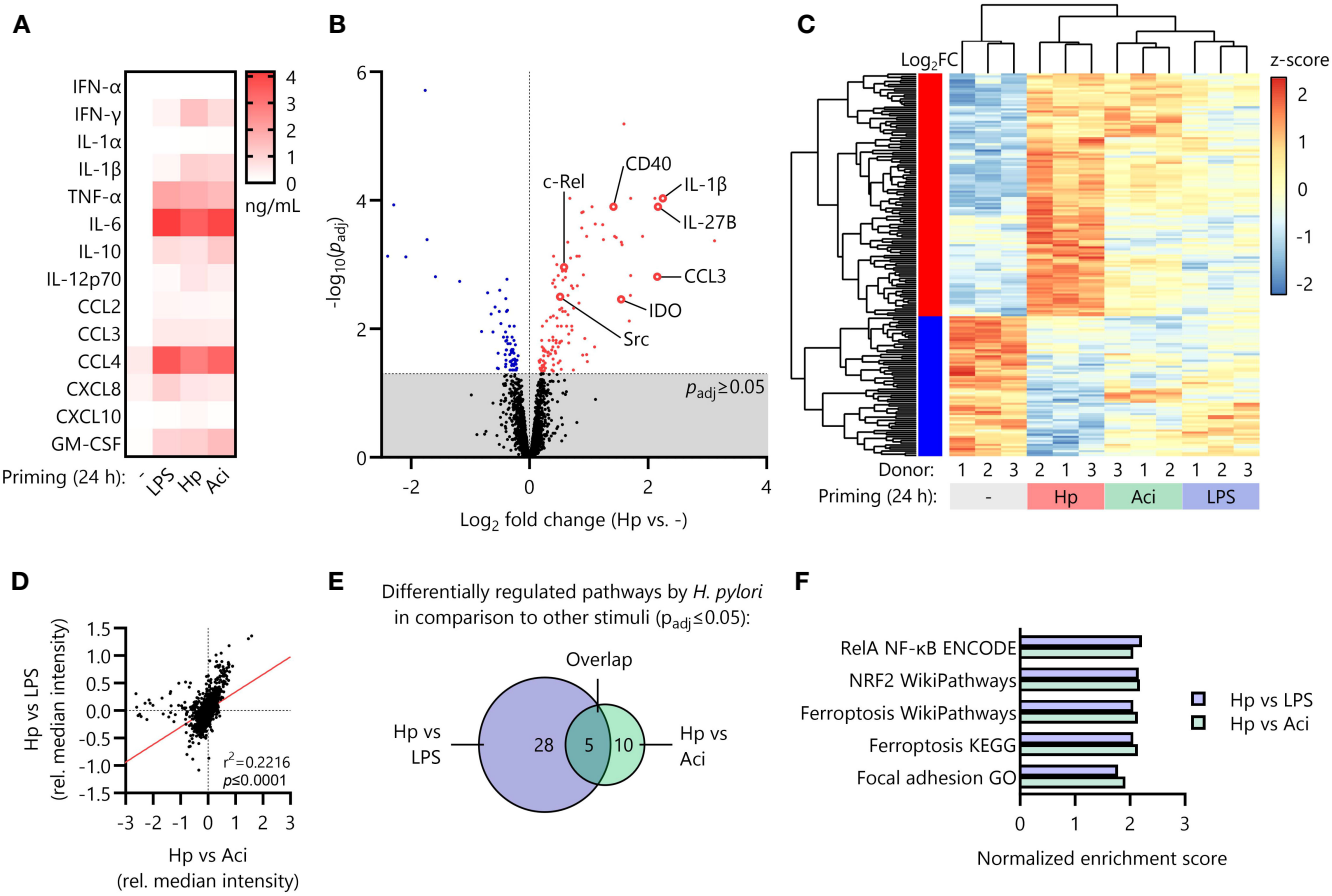
Distinct proteomic signature induced by *H. pylori* in comparison to other priming stimuli. **(A)** Monocytes were primed with LPS, *H*. *pylori* (Hp), *A*. *lwoffii* (Aci) or remained uninduced for 24 (h) Cyto- and chemokine secretion was quantified via Luminex Assay. Heatmap squares indicate mean of four individual donors. **(B)** Volcano plot showing significantly up (red)- and downregulated (blue) proteins in *H*. *pylori*-primed monocytes in comparison to unstimulated controls as analyzed by mass-spectrometry proteomics. Dots represent mean of three individual donors from two independent experiments. **(C)** Comparison of expression levels (z-scores of normalized intensities) of significantly up- or downregulated proteins by *H*. *pylori* in comparison to uninduced controls with levels of those in *A*. *lwoffii*- and LPS-primed cells. **(D)** Correlation of comparisons between *H*. *pylori* and LPS and *H*. *pylori* and *A*. *lwoffii*. **(E)** Number of significantly differentially regulated pathways between *H*. *pylori*- and LPS-primed monocytes, between *H*. *pylori*- and *A*. *lwoffii*-primed monocytes, and the number of overlapping pathways. **(F)** Significantly regulated pathways in both *H*. *pylori*- vs LPS-, and *H*. *pylori*- vs. *A*. *lwoffii*-primed monocytes.

It is well-known that certain pro- and anti-inflammatory cytokines (e.g., TNF-α and IL-10) modulate cellular responsiveness to subsequent stimuli and induce tolerization of immune cells (17, 30). We therefore initially measured secretion of pro- and anti-inflammatory cytokines as well as chemotactic molecules secreted during the priming phase (24 h) via Luminex assay. However, we found that the secretory profiles for all priming stimuli tested (LPS, Hp, Aci and uninduced controls) were decidedly similar and thus not likely to be responsible for the specific priming effect of *H. pylori* (**Figure 2A**).

Reasoning that the intracellular protein composition after the initial priming phase (24 h) may contribute to the LPS-hyperresponsiveness observed upon *H. pylori* infection, we next performed whole-cell proteomic analysis of primed monocytes. Principal component analysis of the proteomic data revealed strong clustering of individual donors corresponding to priming stimuli (**Supplementary Figure S2**). Investigation of differential regulation between unstimulated controls and *H. pylori*-infected monocytes revealed 187 significantly regulated proteins, including upregulation of well-known pro-inflammatory mediators such as interleukin-1α (IL1A) and -1β (IL1B), the co-stimulatory feedback receptor CD40 and NF-кB protein family members (REL, NFKB1, NFKB2), but also anti-inflammatory molecules and enzymes such as indoleamine-deaminase (IDO) (**Figure 2B**).

The expression intensities of the 187 significantly up- or downregulated proteins in *H. pylori*-stimulated cells differed, often greatly so, from the expression levels of those same proteins in LPS- and *A. lwoffii*-stimulated monocytes (**Figure 2C**).

This observation is further corroborated by correlating the relative protein expression of *H. pylori*-primed monocytes compared to LPS-primed or *A. lwoffii*-primed cells, which reveals a highly significant positive association (**Figure 2D**).

To distill the multitude of observed protein regulations down to separate hypo- or hyper-active pathways, we performed fast gene set enrichment analysis as described previously (27). Notably, no pathway was significantly differentially regulated between LPS-stimulated and *A. lwoffii*-stimulated monocytes, underscoring the resemblance of responses to these two priming stimuli. When comparing the differentially regulated pathways (both up- and down-regulated) in *H. pylori* and LPS-stimulated monocytes, the components of a total of 28 pathways were significantly enriched. Pathway analysis comparing *H. pylori*- and *A. lwoffii*-stimulated monocytes revealed 10 pathways being differentially regulated (**Figure 2E**). Remarkably, 5 pathways were upregulated in both comparisons, which included the NRF2 pathway, the RelA NF-кB pathway, ferroptosis and focal adhesion (**Figures 2E, F**). This set of data highlights the unique proteomic signature induced by priming human monocytes with *H. pylori* compared to other tested stimuli.

### The NRF2 pathway does not influence hyperresponsiveness after *H. pylori* priming

**Figure 3:**
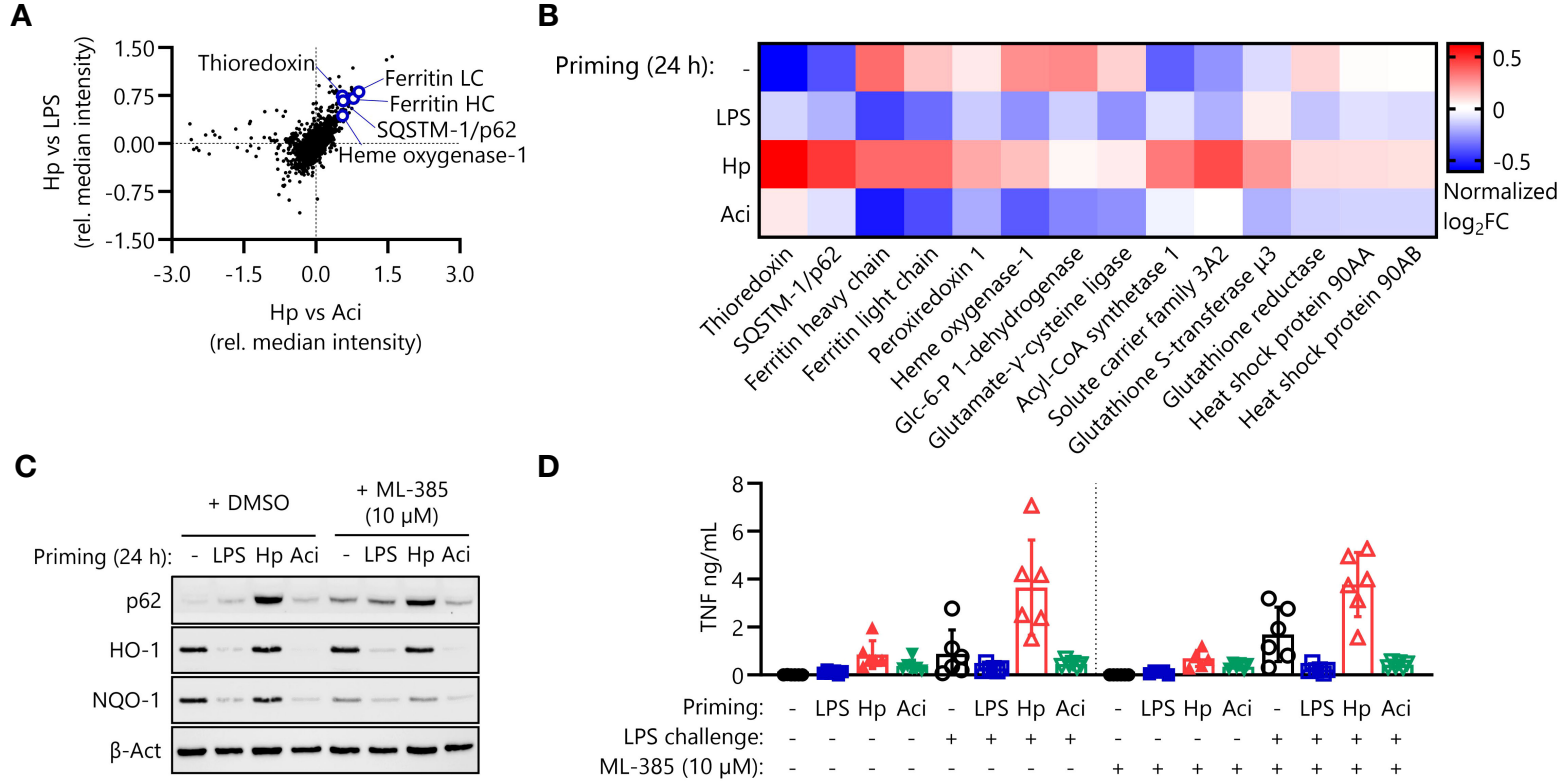
The NRF2 pathway is active in *H. pylori*-primed monocytes but does not drive LPS hyperresponsiveness. **(A)** NRF2 pathway proteins are upregulated in *H*. *pylori*-primed monocytes in comparison to both LPS and *A*. *lwoffii*-primed cells as measured by mass spectrometry proteomics. **(B)** Proteomic expression patterns of NRF2 pathway-associated proteins in cells primed with LPS, *H*. *pylori* (Hp), *A*. *lwoffii* **(Aci)** and uninduced controls. Heatmap squares correspond to mean relative expression of three individual donors. **(C)** Immunoblot showing expression of p62/SQSTM-1, HO-1, NQO-1 and β-actin (loading control) in cells primed with LPS, *H*. *pylori*, *A*. *lwoffii* and uninduced controls treated with DMSO (solvent control) or NRF2 inhibitor ML-385 (10 µM). One representative donor out of two from one experiment is shown. **(D)** TNF-α secretion by monocytes primed with LPS (5 ng/mL), *H*. *pylori*, *A*. *lwoffii* (both MOI 5) or unprimed cells, in presence or absence (DMSO used as solvent control) of NRF2 inhibitor ML-385 (10 µM) during priming, and challenged, or not, with LPS (10 ng/mL) was measured via ELISA. Bars represent mean ± SD of six individual donors and two independent experiments.

We first focused our attention on the effects of the nuclear factor erythroid 2-related factor 2 (NRF2) pathway, which is involved in the intracellular anti-oxidative stress response (**Figure 2F**). Proteomic analysis indicates upregulation of a variety of transcriptional NRF2 targets in *H. pylori*-primed monocytes in comparison to both LPS- and *A. lwoffii*-primed cells (**Figures 3A, B**). Western blot analysis confirms the finding that the expression of NRF2 targets is increased or remains high in *H. pylori* priming compared to downregulation upon treatment with other priming stimuli (**Figure 3C**, left panel). We next sought to assess the impact of NRF2 signaling on post-priming hyperresponsiveness of *H. pylori*-stimulated monocytes. To that end, we inhibited the pathway during the 24 h of priming by adding the NRF2 inhibitor ML-385 (10 µM) 30 minutes prior to infection. The effects of NRF2 inhibition were assessed via Western blot analysis of protein expression changes. Indeed, expression of the NRF2 target NQO-1 was downregulated in ML-385-treated monocytes, whereas the expression of HO-1, another putative transcriptional target of NRF2, remained unaffected (**Figure 3C**, right panel). NRF2 inhibition, however, did not influence or impede the induction of *H. pylori*-mediated hyperresponsiveness to LPS challenge (**Figure 3D**).

### Amplification of NF-кB signaling by *H. pylori* enables hyperresponsiveness to LPS

**Figure 4:**
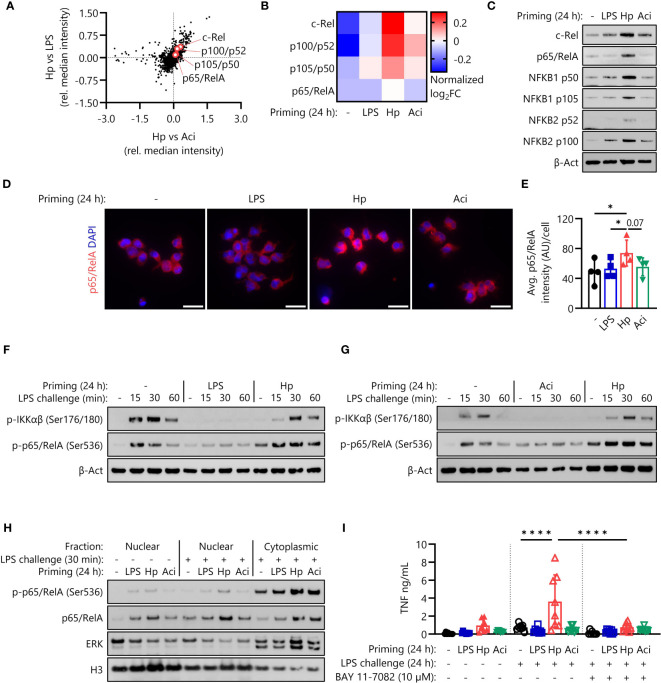
*H. pylori* priming increases intracellular abundance of NF-кB proteins, amplifying subsequent inflammatory responses. **(A)** NF-кB family proteins are upregulated in *H*. *pylori*-primed monocytes in comparison to both LPS- and *A*. *lwoffii*-primed cells as measured by mass spectrometry proteomics. **(B)** Proteomic expression patterns of NF-кB family proteins in monocytes primed with LPS, *H*. *pylori*, *A*. *lwoffii* and uninduced controls. Heatmap squares correspond to relative expression means of three individual donors from two independent experiments. **(C)** Immunoblots showing enhanced expression of NF-кB subunits c-Rel, p65 (RelA), p105/p50 (NF-кB1) p100/p52 (NF-кB2) and β-actin (loading control) of monocytes primed with *H*. *pylori* in comparison to cells primed with LPS, *A*. *lwoffii* or uninduced controls. One representative donor out of six from three independent experiments is shown. **(D)** Immunofluorescence staining for total intracellular p65/RelA in monocytes primed with LPS, *H*. *pylori*, *A*. *Iwoffii* or uninduced controls. DAPI was used to visualize cell nuclei. Scale bar: 20 µm. Cells from one individual donor out of four from two independent experiments are shown. **(E)** Quantification of p65/RelA intensity from **(D)** relative to cell number. Bars represent mean ± SD of four individual donors and two independent experiments. For statistical analysis, RM-ANOVA with Šidak’s *post-hoc* test was performed (* *p* ≤ 0.05) **(F, G)** Immunoblots showing expression of both phosphorylated IKKαβ and p65/RelA, indicating active NF-кB signaling in naïve and *H*. *pylori*-primed monocytes, while being absent in LPS- and *A*. *lwoffii*-primed monocytes. One representative donor of four donors from two independent experiments is shown, respectively. **(H)** Immunoblots showing nuclear and cytoplasmic expression of phosphorylated (Ser536) and total p65/RelA levels in human monocytes, indicating both increased presence and translocation upon challenge in *H*. *pylori*-primed cells in comparison to naïve as well as LPS- and *A*. *lwoffii*-primed controls. Expression of histone H3 and ERK1/2 was used as nuclear and cytoplasmic housekeeping control, respectively. One representative donor out of two from one experiment is shown. **(I)** TNF-α secretion of monocytes primed with LPS (5 ng/mL), *H*. *pylori*, *A*. *lwoffii* (both MOI 5) or unprimed cells challenged, or not, with LPS (10 ng/mL) in the presence or absence (in latter case DMSO was used as a solvent control) of NF-кB inhibitor BAY 11-7082 (10 µM) was measured via ELISA. Bars represent mean ± SD of eight individual donors and three independent experiments. For statistical analysis, RM-ANOVA with Šidak’s *post-hoc* test was performed (****p ≤ 0.0001).

Having established that NRF2 signaling is not essential for the induction of *H. pylori*-mediated monocyte priming and subsequent hyperresponsiveness, we next investigated NF-кB signaling as a potential mainspring of this phenomenon. Proteomic analysis of intracellular proteins revealed that four NF-кB proteins were indeed expressed at elevated levels in monocytes primed with *H. pylori* in comparison to LPS-primed and *A. lwoffii*-primed monocytes (**Figures 4A, B**). These findings were verified by Western blotting, which again showed enhanced expression of NF-кB protein family members in *H. pylori*-primed monocytes after priming for 24 h (**Figure 4C**). Similarly, immunofluorescence microscopy and subsequent quantification confirmed the increased intracellular abundance of p65/RelA (**Figures 4D, E**).

To ascertain if NF-кB proteins are not only overly abundant after *H. pylori* priming, but are still functionally active in primed and subsequently LPS-challenged monocytes, we examined phosphorylation of IKKαβ and p65 (RelA), indicative of active intracellular signal transduction. As expected, *H. pylori*-primed monocytes retained their capacity to induce NF-кB signaling culminating in phosphorylation of p65/RelA, while LPS-primed and *A. lwoffii*-primed cells remained unresponsive to LPS challenge (**Figures 4F, G**). Intriguingly, the levels of phosphorylated p65/RelA in *H. pylori*-primed cells exceeded those in naïve, LPS-challenged cells, which had not yet received any prior stimulus.

As a transcription factor, p65/RelA exerts its pro-inflammatory effects in the nucleus. We thus investigated nuclear protein levels of (phospho-)p65/RelA after priming via immunofluorescence microscopy (**Supplementary Figure S3**) and after priming and 30 minutes of LPS challenge via immunoblotting (**Figure 4H**, **Supplementary Figure S4**). While nuclear levels of p65/RelA directly after priming were similar throughout all stimuli (**Supplementary Figures S3B**, **4**), we could observe elevated nuclear levels, and thus enhanced nuclear translocation, of both phosphorylated (Ser536) and total p65/RelA in *H. pylori*-primed, LPS challenged monocytes in comparison to other priming conditions and unprimed, challenged controls (**Figure 4H**, **Supplementary Figure S4**).

To determine if the increased amplitude of NF-кB signaling during LPS challenge is in fact responsible for the enhanced responsiveness of *H. pylori*-primed monocytes, we challenged primed monocytes in the absence and presence of the NF-кB inhibitor BAY 11-7082 (10 µM). The complete lack of any response to LPS challenge in BAY 11-7082-treated monocytes implies that NF-кB signaling is essential for the LPS hyperresponsiveness of *H. pylori*-primed cells (**Figure 4I**).

## Discussion

Chronic inflammation of *H. pylori*-infected epithelial tissue is a major risk factor for the development of gastric cancer. Infiltrating immune cells contribute to the inflammatory processes by secreting an array of cytokines and chemokines which stimulates additional immune cell influx. Innate immune cells, particularly monocytes, are known to rapidly extravasate from blood vessel to sites of ongoing inflammation and infection (31, 32). Accordingly, monocytes can be found in increased numbers in *H. pylori*-infected murine (33) and human epithelial tissue (3). Activation of monocytes and other myeloid cells can be further regulated by functional adaptations induced by previous encounters with various priming agents, and these adaptations can lead to both a reduction as well as an increase in inflammatory potential of an immune cell – referred to as tolerance and training, respectively (34, 35). Priming stimuli have diverse origins and modes of action. The most prominent and well-studied inducer of innate immune tolerance is endotoxin (i.e., LPS, a constituent of the cell wall of Gram-negative bacteria) (36). Stimulation of macrophages with endotoxin leads to transcriptional repression of various pro-inflammatory gene loci, effectively shutting down transcription of cytokines such as TNF-α or IL-6 in response to subsequent challenge stimuli (37). Recently, other mechanisms of tolerance induction, involving rapid shifts in the availability of intracellular signaling molecules, have added to the understanding of tolerance and training (38). Although the induction of such innate immune memory phenotypes is highly context- and concentration-dependent (39), with one recent study showing induction of trained immunity by LPS in alveolar macrophages (40), tolerance is the more common memory program induced in innate immune cells by strong pro-inflammatory priming stimuli, e.g., pathogen-associated molecular patterns (PAMPs, such as TLR ligands) or bacterial organisms (21, 41, 42).

We previously described the startling observation that infection of primary human monocytes with *H. pylori*, rather than inducing immediate tolerance, promotes responsiveness to subsequent LPS challenge (20). Here we report that the hyperresponsiveness to LPS challenge after *H. pylori* priming is not a general adaptation elicited by Gram-negative bacteria, as *A. lwoffii* infection of monocytes results in robust tolerance to a subsequent stimulus. However, none of the well-studied virulence factors associated with *H. pylori*’s subjugation of immunity seem to be directly involved (**Figure 1**).

The secretion of pro-inflammatory cytokines by innate immune cells has profound effects on virtually all cell types, and investigation of TNF-α-induced tolerance in human primary monocytes/macrophages (17) has convincingly revealed the tolerizing effects of this acute-phase cytokine on the reactivity of these cells to subsequent stimuli. Similarly, interleukin-10, the prototypical anti-inflammatory cytokine, is a pivotal player in the induction of endotoxin tolerance and the tolerant macrophage phenotype (30, 43). In our experimental setting, however, none of the tested priming stimuli incited uniquely high levels of cyto- and chemokines (**Figure 2A**) that could indicate a potential role in the induction of innate immune memory.

In contrast to the observed uniformity in secretory activity, proteomic analysis of intracellular protein expression of primed human monocytes revealed significant differences in the responses to different priming stimuli. Notably, the NRF2 and NF-кB signaling pathways and their constituent proteins seem to be significantly upregulated in *H. pylori*-primed monocytes in comparison to LPS- and *A. lwoffii*-primed cells (**Figure 2**). Yet, further experiments established that NRF2 and its transcriptional activity are apparently dispensable for *H. pylori*-induced hyperresponsiveness (**Figure 3**), while functional NF-кB signaling is necessary (**Figure 4**).

Until recently it was thought that phenomena such as endotoxin tolerance and trained immunity predominantly depend on metabolic reprogramming and epigenetic regulation of pro-inflammatory gene promoter accessibility to induce transient silencing (or long-lasting “priming”) of these genes (14, 37, 44–46). New insights have led to the postulation that fluctuations in abundance of intracellular signaling proteins may represent an additional form of acute innate immune memory (38). Wang and colleagues demonstrated, in models of acute memory formation, that dynamic remodeling of the NF-кB signaling network reflecting prior stimulation has profound effects on a cell’s capacity to respond to subsequent stimulation (38). Their study revealed that LPS stimulation (and to a lesser extent stimulation with IL-1β and TNF-α) has a direct negative effect on the signaling capacities of cells in response to subsequent challenge. The described malleability of the NF-кB response, a result of limited IRAK1 availability among other factors, may complement other previously documented mechanisms of innate immune memory formation and endotoxin tolerance. While trained immunity development per se is a relatively time-consuming process, these shifts in intracellular signaling molecule availability may permit cells to adapt more rapidly after receiving a priming stimulus.

NF-кB protein family members (c-Rel, RelA, RelB, p100/p52 and p105/p50) are central regulators of pro-inflammatory processes in innate immune cells, and their activity must be tightly constrained to avoid uncontrolled inflammation. Thus, regulation of expression and post-translational modifications conferring activity (e.g., phosphorylation) is paramount in situations of repeated or continuous exposure. In the present study, we report the accumulation of several NF-кB proteins after *H. pylori* priming – a process which does not seem to be induced by other pro-inflammatory stimuli in our analysis, including LPS and the Gram-negative bacterium *A. lwoffii*. In accordance with Wang and colleagues (38), we describe impaired signaling functionality in LPS- and *A. lwoffii*-primed monocytes subjected to subsequent challenge with LPS, as evidenced by absence of phosphorylated IKKαβ and phosphorylated p65/RelA in challenged cells primed with the aforementioned stimuli. *H. pylori*-primed monocytes, however, show fully functional activation of IKKαβ as well as increased levels of phosphorylated p65/RelA in response to LPS challenge compared to naïve control cells. Similarly, nuclear translocation of p65/RelA in challenged monocytes was increased after priming with *H. pylori.* These results, and the observed abrogation of LPS hypersensitivity upon BAY 11-7082 treatment, indicate that *H. pylori* priming of monocytes increases the intracellular abundance and enhances nuclear translocation of NF-кB proteins, thereby allowing cells to respond more vigorously to subsequent challenge stimuli. Intriguingly, it has recently been shown that NF-кB dynamics and oscillations in turn influence epigenomic reprogramming of macrophages in response to different stimuli (47), highlighting the interconnectedness of different forms of acute and long-term innate immune memory.

**Figure 5:**
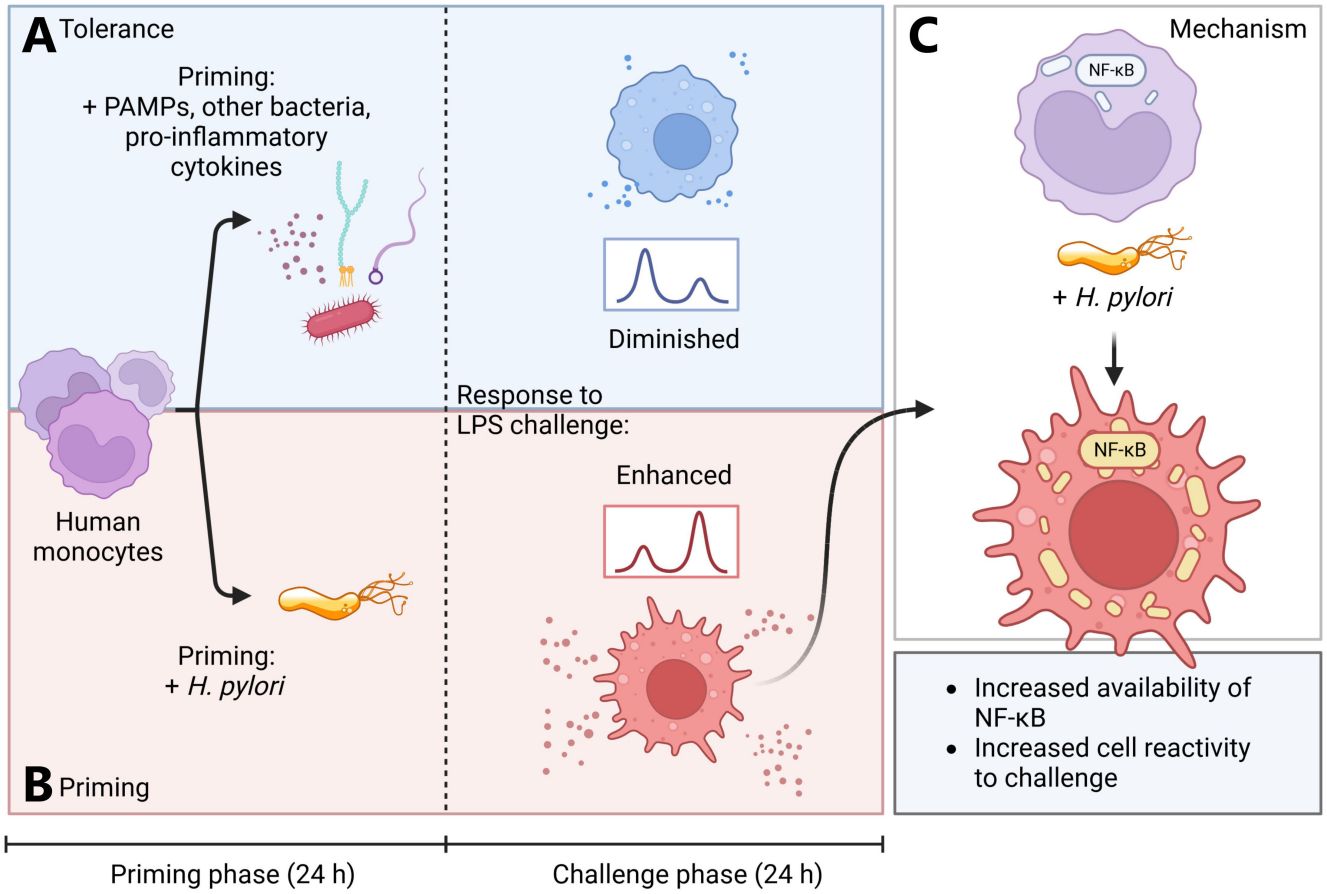
Simplified model of *H. pylori*-induced monocyte priming. (**A**, blue panel) Human monocytes primed for 24 h with various pathogen-associated molecules, *Acinetobacter lwoffii* or pro-inflammatory cytokines (TNF-α) rapidly develop tolerance. (**B**, red panel) *H. pylori*-primed monocytes, however, develop a hyperresponsive phenotype, leading to increased activation upon subsequent LPS challenge. **(C)** This hyperresponsive phenotype is induced by increased abundance of NF-кB subunits and amplified NF-кB signaling in *H. pylori*-primed monocytes. Created with BioRender.com.

Investigation of these mechanisms in experimental settings that better replicate the complex cellular and supracellular composition of the gastric mucosa would be a valuable addition to our growing understanding of *H. pylori*-mediated immunopathology, as numerous reports have recently shed light on the inflammatory plasticity of epithelial and stromal cells (48–50). Our analysis describes opposite forms of innate immune memory, both potentiating and negating subsequent immune responses: *H. pylori*-primed monocytes show enhanced functional NF-кB signaling, whereas LPS- and *A. lwoffii*-tolerized cells effectively shut down the pathway (**Figure 5**). The resulting overactivation of *H. pylori*-primed monocytes leads to increased cytokine responses to subsequent LPS challenge. This hyperactivity could contribute to gastric pathology and inflammation in the context of *H. pylori* infection. The spike in innate immune cell reactivity after contact with the pathogen could also concurrently contribute to the disappearance of other bacterial species from the gastric compartment in presence of the *H. pylori*: several reports have shown dramatic gastric microbiota composition changes upon infection with *H. pylori*, as the microbial diversity of the stomach severely decreases in *H. pylori*-positive individuals (3, 51, 52). Intriguingly, Ferreira and colleagues reported that dysbiosis and loss of microbial richness was even more pronounced in gastric carcinoma samples (53), albeit the relative abundance of *H. pylori* decreased, implying that the damage may have been done. These findings indicate that the degradation of the microbiota composition may play a pivotal role in the etiology of gastric carcinomas. Taken together, both the inflammatory pathology linked to *H. pylori* as well as the significant loss in microbial diversity may be associated with or further exacerbated by the pathogen’s priming effect on monocytes and possibly other innate immune cells.

## Data availability statement

The data presented in the study are deposited in the PRIDE repository (https://www.ebi.ac.uk/pride/archive), accession number PXD045072.

## Ethics statement

Ethical approval was not required for the studies involving humans because this study was conducted in accordance with established guidelines of the World Medical Association’s Declaration of Helsinki. As Austrian national regulations do not require additional consent from anonymous blood donors for scientific use of blood cells discarded after leukapheresis, no further approval of the study by the local ethics committee was required. The studies were conducted in accordance with the local legislation and institutional requirements. The human samples used in this study were acquired from a by- product of routine care or industry. Written informed consent to participate in this study was not required from the participants or the participants’ legal guardians/next of kin in accordance with the national legislation and the institutional requirements.

## Author contributions

TF: Conceptualization, Data curation, Formal Analysis, Investigation, Visualization, Writing – original draft, Writing – review & editing. TN: Conceptualization, Supervision, Writing – review & editing. CR: Data curation, Formal Analysis, Investigation, Writing – review & editing. VS: Data curation, Formal Analysis, Investigation, Visualization, Writing – review & editing. A-LO: Investigation, Writing – review & editing. H-HD: Investigation, Writing – review & editing. MU: Formal Analysis, Investigation, Visualization, Writing – review & editing. CH: Resources, Writing – review & editing. FA: Supervision, Project administration, Funding acquisition, Writing – review & editing. SW: Methodology, Resources, Writing – review & editing. JH-H: Conceptualization, Funding acquisition, Methodology, Resources, Supervision, Validation, Visualization, Writing – review & editing.

## Supplementary material

Supplementary Figure 1 Induction of tolerance and cross-tolerance by priming with various microbial patterns or a pro-inflammatory cytokine. Monocytes were primed with *E. coli* LPS (5 ng/mL) **(A)**, flagellin (FLA) from *Pseudomonas aeruginosa* (50 ng/mL) **(B, C)** or human recombinant TNF-α (5 ng/mL) **(D)** or remained unstimulated for 24 h. Then the medium was exchanged to challenge medium, either containing LPS (10 ng/mL) **(A, C, D)**, FLA (100 ng/mL) **(B)** or no stimulus for controls. TNF-α **(A–C)** or interleukin-6 (IL-6) **(D)** secretion was measured 24 h after challenge via ELISA. Bars represent mean ± SD of three to seven individual donors and one or more independent experiments. For statistical analysis, RM-ANOVA with Šidak’s *post-hoc* test was performed (**p* ≤ 0.05, ****p* ≤ 0.001, *****p* ≤ 0.0001).

Supplementary Figure 2 Principal component analysis reveals strong clustering of individual priming stimuli. Principal component analysis of normalized reporter ion intensities calculated after removal of donor batch effects by *limma* plugin in PerseusR.

Supplementary Figure 3 Similar nuclear p65/RelA levels throughout all priming stimuli after 24 h. **(A)** Immunofluorescence staining for p65/RelA in monocytes primed with *E. coli* LPS (5 ng/mL), *H. pylori*, *A. Iwoffii* (both MOI 5) for 24 h or uninduced controls. DAPI was used to visualize cell nuclei. Scale bar: 10 µm. Cells from one individual donor out of four from two independent experiments are shown. **(B)** Quantification of co-localized signals for p65/RelA and DAPI from **(A)** as a measure of nuclear p65/RelA. Bars represent mean ± SD.

Supplementary Figure 4 Relative quantification of nuclear phospho- and total p65/RelA. **(A)** Immunoblots showing nuclear and cytoplasmic expression of phosphorylated (Ser536) and total p65/RelA levels in human monocytes after priming and ± LPS challenge (donor 2, complementing Main ). Expression of histone H3 and ERK1/2 was used as nuclear and cytoplasmic housekeeping control, respectively. **(B)** Relative nuclear phospho-p65/RelA levels and relative total nuclear p65/RelA levels **(C)** of monocytes primed for 24 h with either LPS (5 ng/mL), *H. pylori*, *A. lwoffii* (both MOI5) or uninduced controls either challenged for 30 min with LPS (10 ng/mL), or not. Relative expression was calculated by normalizing band intensity with nuclear H3 band intensity. Bars represent mean ± SD of two individual donors and one experiment. For statistical analysis, RM-ANOVA with Šidak’s *post-hoc* test was performed (**p* ≤ 0.05, ***p* ≤ 0.01).
