# Supplementary figures and images for: Helicobacter pylori induces a novel form of innate immune memory via accumulation of NF-кB proteins
Source: Front Immunol. 2023 Nov 20;14:1290833. doi: 10.3389/fimmu.2023.1290833 (PMC10694194; doi:10.3389/fimmu.2023.1290833)

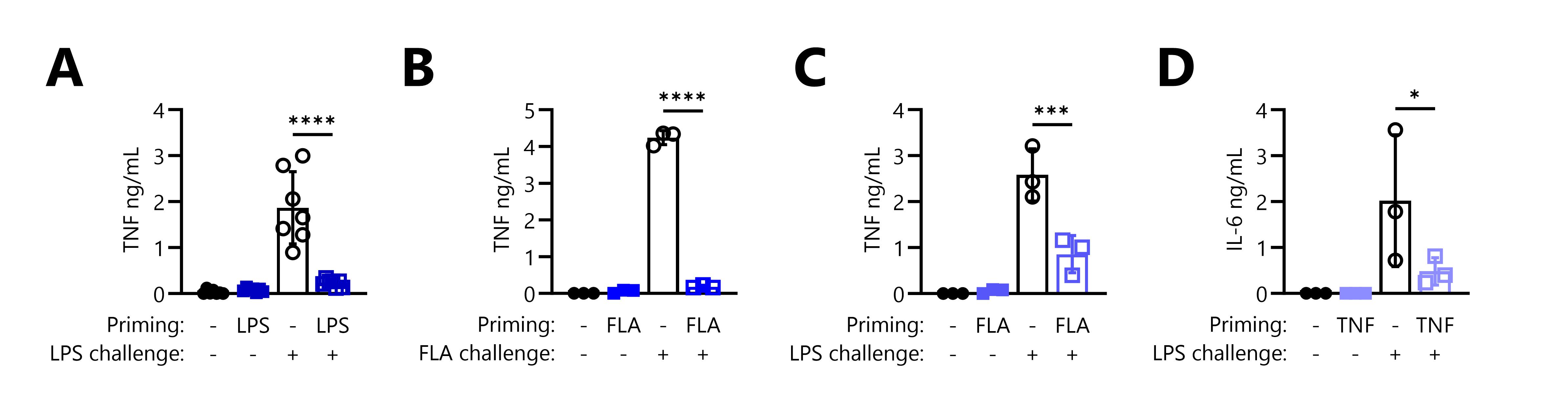

Supplement: Supplementary Figure 1 — Induction of tolerance and cross-tolerance by priming with various microbial patterns or a pro-inflammatory cytokine. Monocytes were primed with E. coli LPS (5 ng/mL) (A), flagellin (FLA) from Pseudomonas aeruginosa (50 ng/mL) (B, C) or human recombinant TNF-α (5 ng/mL) (D) or remained unstimulated for 24 h. Then the medium was exchanged to challenge medium, either containing LPS (10 ng/mL) (A, C, D), FLA (100 ng/mL) (B) or no stimulus for controls. TNF-α (A–C) or interleukin-6 (IL-6) (D) secretion was measured 24 h after challenge via ELISA. Bars represent mean ± SD of three to seven individual donors and one or more independent experiments. For statistical analysis, RM-ANOVA with Šidak’s post-hoc test was performed (*p ≤ 0.05, ***p ≤ 0.001, ****p ≤ 0.0001). [file Image_1.jpeg]

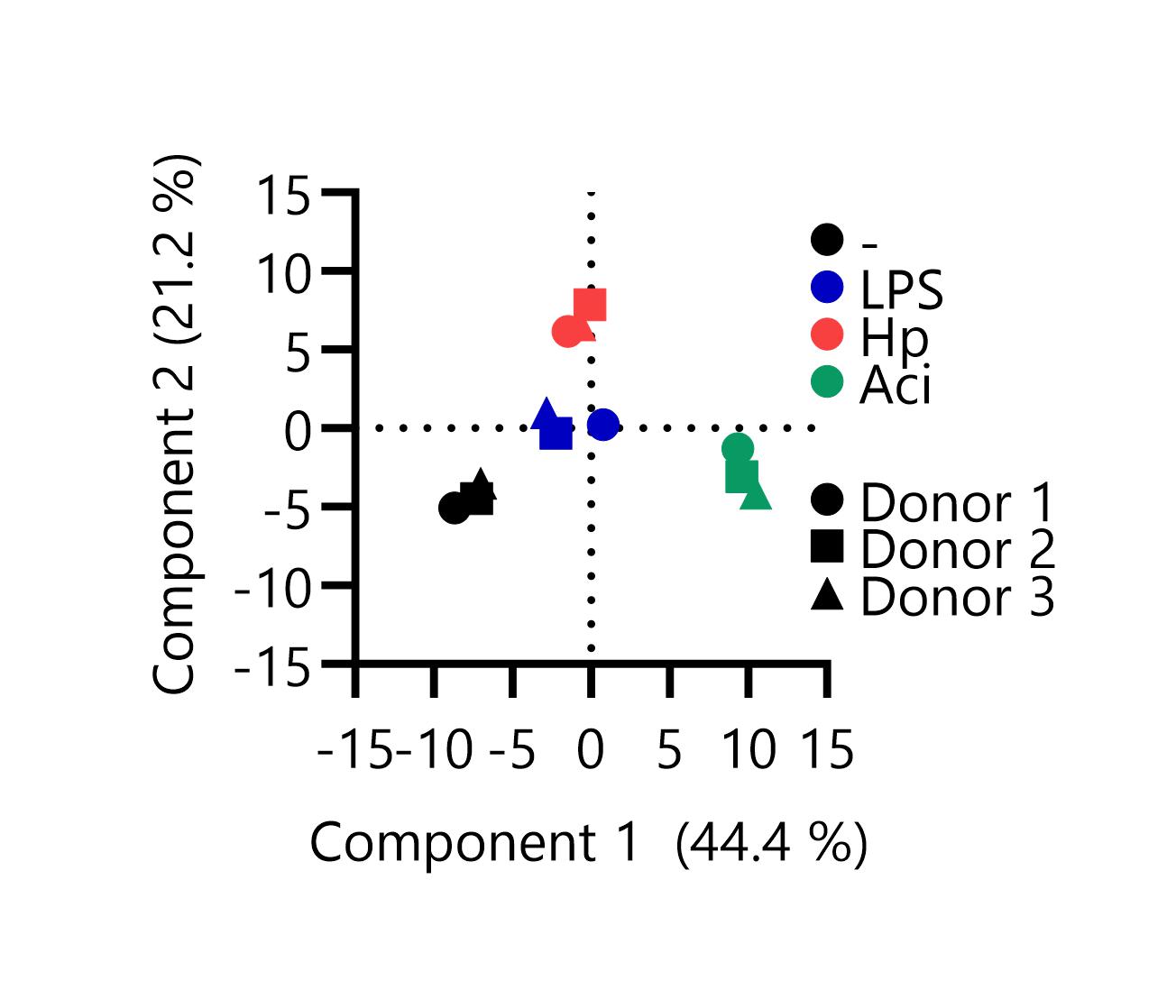

Supplement: Supplementary Figure 2 — Principal component analysis reveals strong clustering of individual priming stimuli. Principal component analysis of normalized reporter ion intensities calculated after removal of donor batch effects by limma plugin in PerseusR. [file Image_2.jpeg]

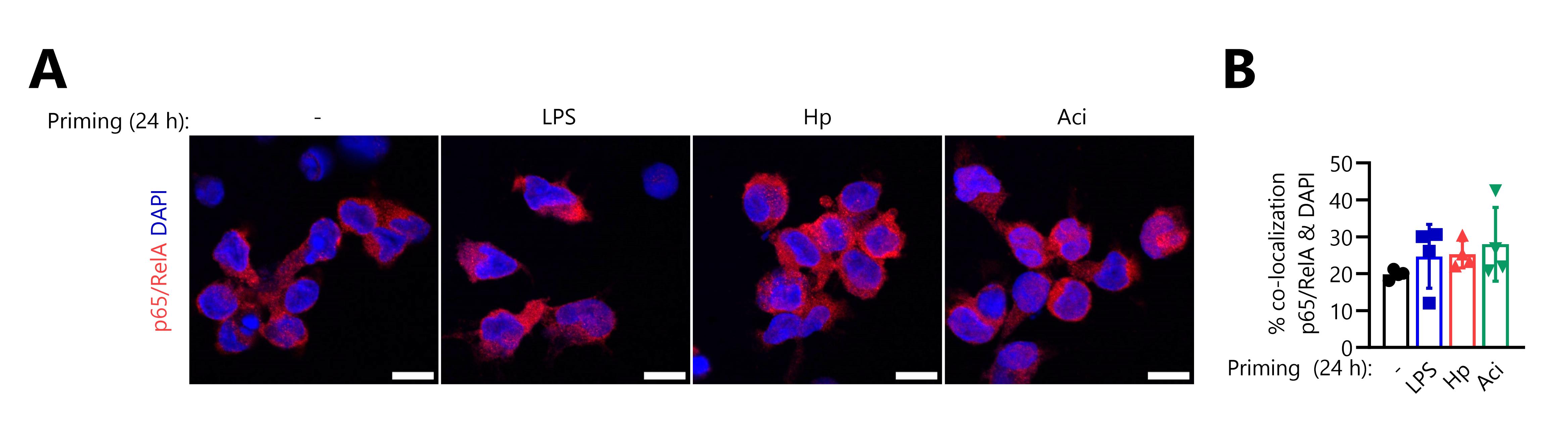

Supplement: Supplementary Figure 3 — Similar nuclear p65/RelA levels throughout all priming stimuli after 24 h. (A) Immunofluorescence staining for p65/RelA in monocytes primed with E. coli LPS (5 ng/mL), H. pylori, A. Iwoffii (both MOI 5) for 24 h or uninduced controls. DAPI was used to visualize cell nuclei. Scale bar: 10 µm. Cells from one individual donor out of four from two independent experiments are shown. (B) Quantification of co-localized signals for p65/RelA and DAPI from (A) as a measure of nuclear p65/RelA. Bars represent mean ± SD. [file Image_3.jpg]

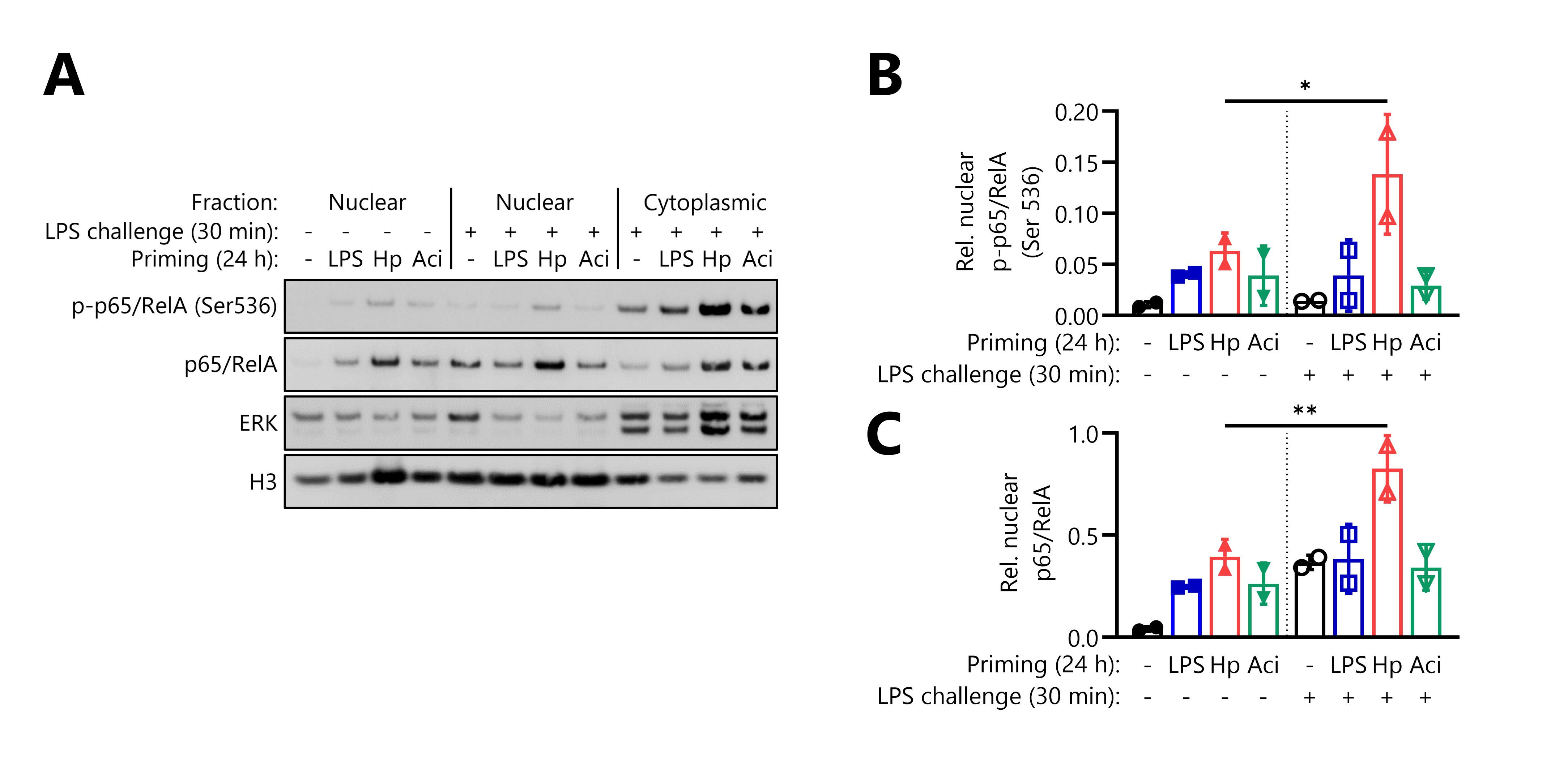

Supplement: Supplementary Figure 4 — Relative quantification of nuclear phospho- and total p65/RelA. (A) Immunoblots showing nuclear and cytoplasmic expression of phosphorylated (Ser536) and total p65/RelA levels in human monocytes after priming and ± LPS challenge (donor 2, complementing Main ). Expression of histone H3 and ERK1/2 was used as nuclear and cytoplasmic housekeeping control, respectively. (B) Relative nuclear phospho-p65/RelA levels and relative total nuclear p65/RelA levels (C) of monocytes primed for 24 h with either LPS (5 ng/mL), H. pylori, A. lwoffii (both MOI5) or uninduced controls either challenged for 30 min with LPS (10 ng/mL), or not. Relative expression was calculated by normalizing band intensity with nuclear H3 band intensity. Bars represent mean ± SD of two individual donors and one experiment. For statistical analysis, RM-ANOVA with Šidak’s post-hoc test was performed (*p ≤ 0.05, **p ≤ 0.01). [file Image_4.jpg]
